# Hyperglycaemic hyperosmolar state in an obese prepubertal girl with type 2 diabetes: case report and critical approach to diagnosis and therapy

**DOI:** 10.1186/s13052-021-00983-z

**Published:** 2021-02-18

**Authors:** Angelika Mohn, Nella Polidori, Valeria Castorani, Laura Comegna, Cosimo Giannini, Francesco Chiarelli, Annalisa Blasetti

**Affiliations:** Department of Paediatrics, University of Chieti, Via dei Vestini, 5, IT-66100 Chieti, Italy

**Keywords:** Hyperglycaemic hyperosmolar state, Obese children, Type 2 Diabetes, Diabetic Ketoacidosis, Case Report

## Abstract

**Introduction:**

Isolated Hyperosmolar Hyperglycaemic Syndrome (HHS) is a life-threatening condition characterized by elevated serum glucose concentrations and hyperosmolality without significant ketosis. It is often described in obese adults with unknown Type 2 Diabetes (T2D), rarely in youth. In childhood the most common cause of metabolic glucose related derangement is Diabetic Ketoacidosis (DKA) in Type 1 Diabetes (T1D). Interestingly, both components can be combined with each other, thus the prevalent condition needs to be recognised implying a different therapeutic approach.

**Case presentation:**

In this case, we report a prepubertal Caucasian obese girl admitted for two episodes of combined HHS/DKA in order to elucidate her clinical course taking into account the current pediatric recommendations based on adult guidelines for HHS.

**Conclusions:**

The treatment of HHS and even more of HHS/DKA in youth is still controversial as no specific guidelines for children are available especially during the prepubertal age. The description of our case might be helpful and offer relevant points for future consensus.

## Introduction

Recent trends indicate a rising in the incidence of Type 2 Diabetes (T2D) in children, with a significant increase in morbidity and its related complications [1–3]. Hyperosmolar Hyperglycaemic Syndrome (HHS) is the most common and most relevant metabolic consequence described in adults with T2D [4–7]. However, there are few reports of HHS in adolescents with newly diagnosed T2D while no reports are available in prepubertal subjects. Due to the high mortality rate and severe complications associated with HHS, it is imperative to distinguish HHS from the most common Diabetic Ketoacidosis (DKA) in childhood since erroneous clinical management might highly influence clinical outcomes [2, 5–9].

Hereby we report a prepubertal Caucasian obese girl admitted for two episodes of combined HHS/DKA in order to elucidate her clinical course taking into account the current pediatric recommendations based on adult guidelines for HHS.

## Case report

A prepubertal, Caucasian 11 years-old girl was admitted to the Pediatric Department of Chieti for evaluation of severe hypertension (200/114 mmHg) in the context of severe obesity [Weight: 86.9 kg, Height: 144 cm, BMI: 41.9 kg/m^2^ (SDS-BMI: 3.25)]. She had a family history of obesity (both parents and the 17 years-old brother) and both parents were affected by T2D. The girl had a normal weight at birth, developed obesity since the age of 5 years-old. Clinical examination revealed marked acanthosis nigricans and striae rubrae. Essential Hypertension was diagnosed and therapy with amlodipine and bisoprololo was successfully started. The girl presented normal glucose metabolism (fasting glycaemia: 79 mg/dL, HbA1c: 5.4%) with insulin resistance (fasting insulin: 51 mU/mL, HOMA-IR index: 9.9), normal lipid profile (total Cholesterol: 197 mg/dL, HDL: 23 mg/dL, LDL Cholesterol: 137 g/dL, Triglycerides: 185 g/L) and hepatic steatosis with mildly increased transaminases levels (AST: 62 U/L, ALT: 122 U/L). Lifestyle change was recommended including a detailed dietary scheme and physical activity program. Thereafter, during the ambulatory follow-up hypertension was well controlled but the patient was lost after 2 months.

After six months she was admitted to the emergency department [Weight: 78.5 kg, Height: 146 cm, BMI: 36.8 kg/m^2^ (SDS-BMI: 2.97)] for lethargy. She had an history of chest pain associated with increasing dyspnoea and progressive drowsiness over the last two days before presentation. Weight lost and intense polyuria over the 4 weeks was reported.

On admission, Glasgow Coma Scale (GCS) was 13/15, heart rate 120 beats/min, respiratory rate 40 acts/min, blood pressure 140/70 mmHg and pulse oximetry 100% on room air. At clinical evaluation she appeared severely dehydrated. Neurological examination confirmed profound drowsiness although awakeable, with eye opening response to speech and mild confused verbal response. She denied headache and no neurological alterations were reported.

**Fig. 1:**
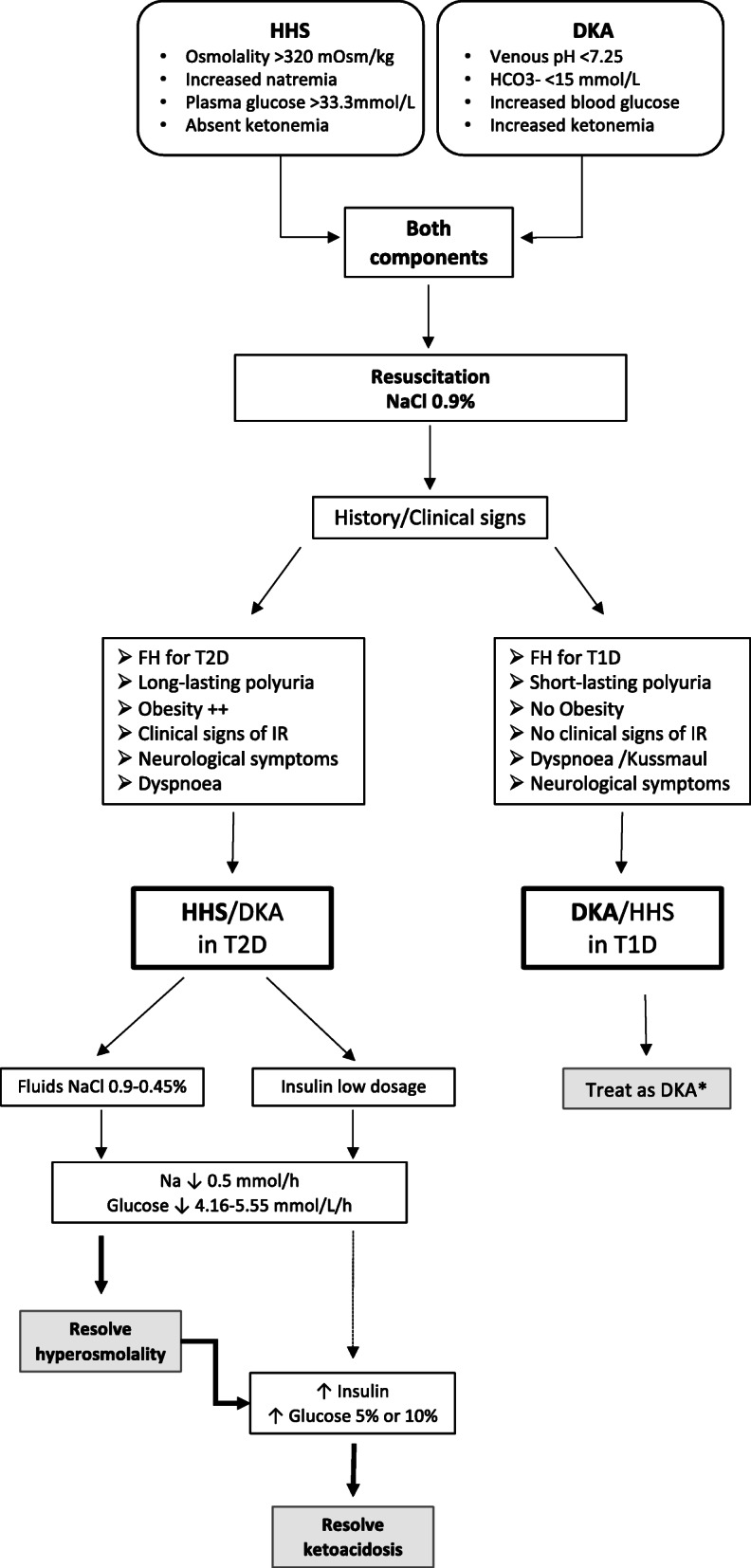
Algorithm of combined HHS/DKA status in children with T2D. FH, *Family History.* *Wolfsdorf JI, Glaser N, Agus M et al. ISPAD Clinical Practice Consensus Guidelines 2018:Diabetic Ketoacidosis and Hyperglycemic Hyperosmolar State. Pediatr Diabetes. 2018;27:155-177

Kidney function (creatinine: 0.70 mg/dL) and potassium levels were normal. In contrast, on blood gas evaluation severe hyperglycaemia (647 mg/dL), increased sodium levels (corrected Na: 152 mmol/L) associated with high serum osmolality (326 mmol/Kg) were detected. In addition, severe acidosis with low bicarbonates and high ketonemia levels (pH: 7.11, BE: -23.9, HCO3-: 9.2 mmol/L, ketonemia >8 mmol/L) were demonstrated establishing a diagnosis of combined HHS and DKA. Clinical history together with well-known insulin resistance state and long lasting polyuria oriented towards metabolic derangement due to unknown T2D. In this respect therapy was started with the aim to control primarily the hyperosmolar state (Fig. 1).

In fact, rehydration with isotonic saline (0.9% NaCl) infusion was initially started and after four hours continued with 0.45% NaCl together with continuous low-dose insulin administration (Table 1). Infusion treatment was stopped after 60 hours when normal serum osmolality and pH levels were achieved, although blood ketones were still present. Therefore, subcutaneous basal-bolus insulin administration was started. Over the infusion period general condition progressively ameliorated and no neurological or other complications were developed.

**Table 1:**
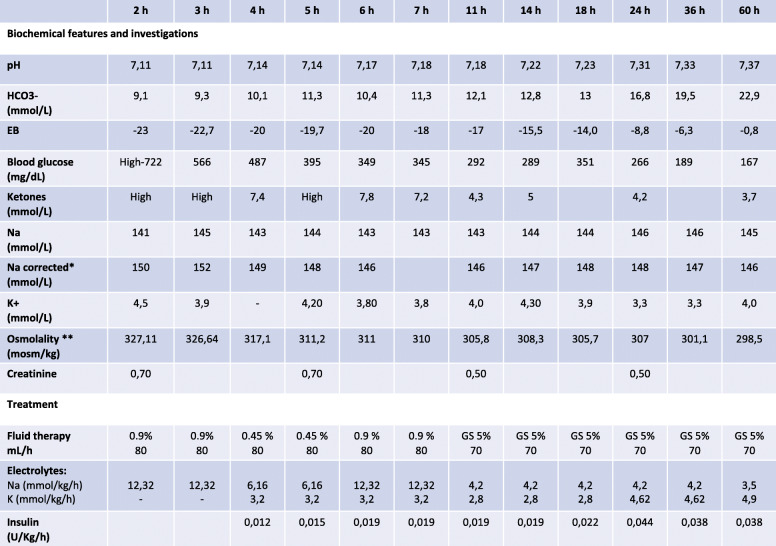
Detailed summary of clinical course in reference to infusion therapy of the first combined HHS/DKA episode

*Corrected sodium formula: Corrected sodium (mmol/L) = measured Na + 2([plasma glucose/100]/100)

**Effective osmolality (mOsm/kg) = 2 x (plasma Na) + plasma glucose mmol/L (normal range is 275 to 295 mOsm/kg)

The suspicion of T2D complicated by combined HHS and DKA, was confirmed by detection of high percentage of HbA1c (11.6%), high levels of C-peptide (3.24 ng/mL) and the undetectable titre of antibodies against pancreatic islets’ antigens (ICA, GAD, IA2) while ZnT8 antibodies were not been performed. She stopped basal-bolus insulin administration after 17 days and continued with degludec therapy once a day and metformin therapy twice daily obtaining normal glucose metabolism after two months (HbA1c: 5.2%). Lifestyle changes were endorsed but after some months the girl was lost for follow-up.

After 2 years, she presented again to the emergency department with a similar episode characterized by increased dyspnoea, nausea, lack of appetite, sporadic vomiting and a poor glycaemic control over the last seven days [Weight: 95.5 kg, Height: 155 cm, BMI: 39.8 kg/m^2^ (SDS-BMI: 3.41), Pubertal stage: P5B5]. Parents reported poor compliance over the last months.

On admission neurological examination was normal (GCS 15/15). She did not complain headache, neither other neurological symptoms. She was severely dehydrated.

Blood tests performed showed again normal potassium values, while kidney function was borderline (creatinine: 1.26 mg/dL, BUN: 16 mg/dL). Blood gas demonstrated hyperglycaemia (402 mg/dL), increased sodium concentrations (corrected Na: 152 mmol/L), associated with mild increased serum osmolality (316.4 mmol/Kg). Moreover, acidosis and ketonemia (pH: 7.23, BE: -18.8, HCO3-: 26.4 mmol/L and ketonemia: 5.6 mmol/L) were documented.

Initial combined HHS/DKA was supposed and isotonic saline (0.9% NaCl) infusion was started first and subsequently substituted by 0.45% NaCl for rehydration (Table 2). After two hours, continuous low dose insulin administration was started and associated thereafter with glucose 5% after 11 hours. Moreover, when osmolality was acceptable but metabolic acidosis with high blood ketone levels persisted after 20 hours, glucose infusion at 10% was started and maintained up to 60 hours post admission (Figure 1) with insulin infusion (0.042 U/kg/h). Normal serum osmolality, pH levels and resolution of ketonemia were achieved. General condition ameliorated progressively and no neurological symptoms appeared.

**Table 2:**
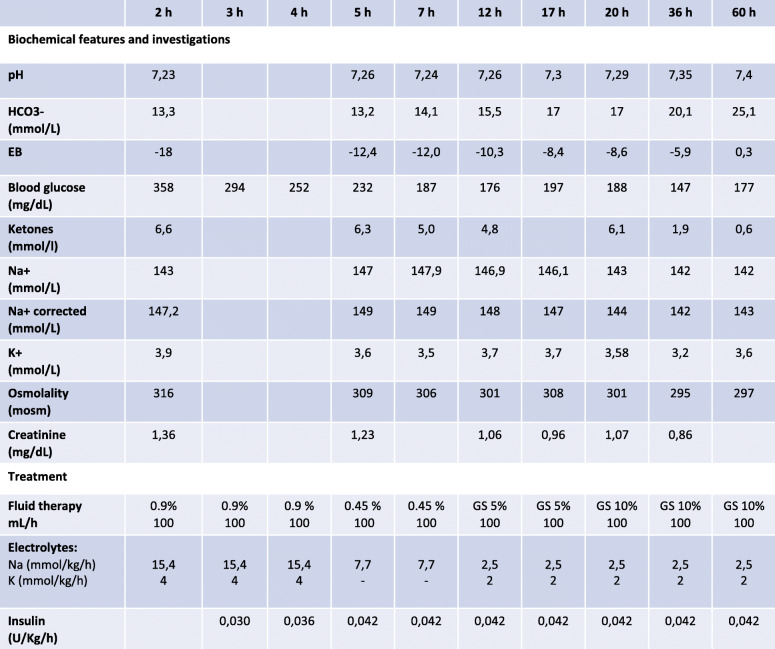
Detailed summary of clinical course in reference to infusion therapy of the second mild combined HHS/DKA episode

Poorly controlled T2D (HbA1c: 11.6%) in the presence of detectable levels of C-peptide (1.64 ng/mL, normal range 0.69-2,45 ng/mL) was found and undetectable titre of antibodies against pancreatic islets’ antigens (ICA, GAD, IA2) were confirmed. Degludec therapy once a day and metformin therapy twice daily was confirmed obtaining good metabolic control.

## Discussion and conclusions

The description of this clinical case highlights the possibility of developing combined HHS/DKA already in the prepubertal age in the context of unknown or uncontrolled T2D. Isolated HHS is a life-threatening condition characterized by elevated serum glucose concentrations and hyperosmolality without significant ketosis [5]. Although this state is well described in obese adults affected by unknown T2D [4, 8], its incidence is estimated to be only 2% of youth and more importantly, to the best of our knowledge, it has not been reported so far in the prepubertal age [6]. In fact, in childhood the most common cause of metabolic glucose related derangement is DKA in newly diagnosed T1D or poorly controlled disease. Interestingly, both components can be combined with each other, thus the prevalent condition needs to be recognised implying a different therapeutic approach [9]. This arduous task can be achieved only thorough an accurate clinical history and examination.

While DKA results from an absolute insulin deficiency, HHS is mainly the results from a relative insulin deficiency. Relative or absolute insulin deficiency leads to increased circulating levels of glucagon and other counter-regulatory hormones. These effects accelerate gluconeogenesis and glycogenolysis and deteriorate peripheral insulin sensitivity leading thereby to a steadily progressive hyperglycaemia. However, compared to DKA the higher hepatic and circulating insulin concentrations present in the HHS in association with lower glucagon levels. Thus, these changes determine a higher circulating ratio of insulin/glucagon preventing ketogenesis and therefore the development of chetoacidosis in HHS. In contrast the ongoing glycosuria in HHS induces persistent osmotic diuresis leading to progressive hypovolemia, intracellular dehydration and eventually reduction in glomerular filtration rate inducing a hyperosmolar state due not only to severe hyperglycaemia but also to the development of hypernatremia. Therefore, HHS takes place over a longer period of time than in patients with DKA, resulting in a more severe state of dehydration, hyperglycaemia, hypernatremia determining profound increase of plasma hyperosmolality, all of which correlate with the always present impaired levels of consciousness and the high rate of mortality.

The underlying mechanisms for the development of HHS/DKA remain to be elucidated although it might be the consequence of a more profound lack of hepatic and peripheral insulin availability with respect to isolated HHS. In fact, our patient presented extreme insulin resistance as documented by the extremely elevated fasting insulin levels six months before and the extreme high levels of C-peptide detected during the first episode. The associated hepatic steatosis a further indicator of insulin resistance might facilitate the development of lipid alterations in terms of lipolysis lying the grounds for ketogenesis and metabolic acidosis [10].

The treatment of HHS and even more of HHS/DKA in youth is still controversial as no specific guidelines for children are available especially during the prepubertal age and the description of our case adds valuable help to current literature.

Replacement of lost fluid is the first critical step in the management of both HHS and DKA. But given the profound degree of dehydration determined over a longer period of time in patients with HHS, aggressive and long lasting fluid therapy with isotonic or hypotonic saline is the basis of treatment. Fluid therapy is crucial in order to expand both intra and extracellular volume, to restore renal perfusion and thereby reduce levels of counter-regulatory hormones and hyperglycaemia. Data show that patients with HHS treated with aggressive fluid therapy had better rates of survival [11]. Isotonic fluids first and hypotonic fluids subsequently are recommended in order to slowly correct the hypernatremia and thereby plasma osmolality. In contrast, after initial resuscitation with aggressive fluid therapy, in DKA hyper-hydration might be an underlying trigger factor for the development of cerebral oedema. Thus, long lasting high volume fluid intake is not recommended during DKA. In this case the underlying cause for the metabolic derangement based on clinical and biochemical elements was HHS complicated by DKA. Resuscitation therapy was not performed since signs and symptoms of cardiovascular shock were not present. High dose infusion of isotonic fluids first and hypotonic fluid subsequently was administered with the aim of restore primarily peripheral circulation and subsequently reach a satisfactory osmolality through a progressive decline of hypernatremia. Low dose insulin therapy was started after three hours, earlier than a reduction of plasma glucose of 50 mg/dL per hour with fluid alone had been obtained. The underlying aim was not only to resolve hyperosmolality through normalization of hyperglycaemia but also to improve her peculiar clinical background characterized by severe insulin resistance. Usually, correcting hyperosmolality is sufficient to correct also a slight metabolic acidosis determined by lactic acidosis in isolated HHS which has not been achieved in this patient. In the present case we reached acceptable osmolality after 36 hours in the first episode and after 20 hours in the second episode maintaining during both episodes an important DKA component. Once hyperosmolality has been restored, all efforts should be placed to concentrate on resolution of the persisting ketoacidosis. In fact, a new steady state between insulin resistance, insulin infusion and glucose consumption has been obtained and glucose fluid therapy can be used in order to obtain complete suppression of lipolysis and ketogenesis. In addition, in our experience glucose infusion at 10% can be safely used although considered a hypertonic solution due to its high glucose content as glucose uptake is guaranteed by the improved insulin resistant state.

In conclusion, HHS/DKA must be considered also in prepubertal children. A two steps isotonic/hypotonic infusion and insulin treatment should be started to correct hyperosmolality first through correction of both hyperglycaemia and hypernatremia. Once near normal glycaemic control has been obtained through satisfactory insulinization, a complete inhibition of ketogenesis can be properly achieved. This arduous task can be achieved only thorough an accurate clinical history, examination and a careful monitoring of young patients with HHS/DKA at high risk of complications.

## Data Availability

Data sharing not applicable to this article as no datasets were generated or analysed during the current study. The data used and/or analysed during the writing of this manuscript are available from the corresponding authors on reasonable request.

## Abbreviations

HHS: Hyperosmolar Hyperglycaemic Syndrome
DKA: Diabetic ketoacidosis
T2D: Type 2 Diabetes
BMI: Body Mass Index
GCS: Glasgow Coma Scale
HbA1c: Glycated Haemoglobin
ICA: Islet Cell Antibodies
GAD: Glutamic Acid Decarboxylase autoantibodies
IA-2: Islet Antigen 2 antibody
